# Phenotypic, Hormonal, and Genomic Variation Among *Vitis vinifera* Clones With Different Cluster Compactness and Reproductive Performance

**DOI:** 10.3389/fpls.2018.01917

**Published:** 2019-01-07

**Authors:** Jérôme Grimplet, Sergio Ibáñez, Elisa Baroja, Javier Tello, Javier Ibáñez

**Affiliations:** Instituto de Ciencias de la Vid y del Vino (CSIC, Universidad de La Rioja, Gobierno de La Rioja), Logroño, Spain

**Keywords:** reproductive performance, grape cluster compactness, fruitset, hormones, phenotyping, somatic variation, transcriptomics

## Abstract

Previous studies showed that the number of berries is a major component of the compactness level of the grapevine clusters. Variation in number of fruits is regulated by events occurring in the fruitset, but also before during the flower formation and pollination, through factors like the initial number of flowers or the gametic viability. Therefore, the identification of the genetic bases of this variation would provide an invaluable knowledge of the grapevine reproductive development and useful tools for managing yield and cluster compactness. We performed the phenotyping of four clones (two compact and two loose clones) of the Tempranillo cultivar with reproducible different levels of cluster compactness over seasons. Measures of reproductive performance included flower number per inflorescence, berry number per cluster, fruitset, coulure, and millerandage indices. Besides, their levels of several hormones during the inflorescence and flower development were determined, and their transcriptomes were evaluated at critical time points (just before the start and at the end of flowering). For some key reproductive traits, like number of berries per cluster and number of seeds per berry, clones bearing loose clusters showed differences with the compact clones and also differed from each other, indicating that each one follows different paths to produce loose clusters. Variation between clones was observed for abscisic acid and gibberellins levels at particular development stages, and differences in GAs could be related to phenotypic differences. Likewise, various changes between clones were found at the transcriptomic level, mostly just before the start of flowering. Several of the differentially expressed genes between one of the loose clones and the compact clones are known to be over-expressed in pollen, and many of them were related to cell wall modification processes or to the phenylpropanoids metabolism. We also found polymorphisms between clones in candidate genes that could be directly involved in the variation of the compactness level.

## Introduction

Cluster compactness, the density of the berries in the cluster, is a primary aspect of grapevine (*Vitis vinifera* L.) selection programs. Berries inside the cluster are organized as a thyrse, which is unique to grapevine among economically important crops. The thyrse, etymologically derived from Dionysus’s staff, is a type of mixed compound inflorescence. It is a raceme inflorescence type where flowers are replaced by a cyme (Hickey and King, 2000; Gerrath et al., 2017), also described as a panicle (Pratt, 1971). Grapevine cluster compactness is an economically important trait since it affects several major component of the fruit quality (reviewed by Tello and Ibáñez, 2018). Foremost, compact clusters are more susceptible to pests and diseases. Several reasons have been pointed out to explain it: first, compact structure favors propagation of pathogen agents within the cluster and lack of aeration among the berries create a suitable environment for their proliferation; besides, berries in close contact to each other showed a reduction of the protective cuticular wax (Marois et al., 1986; Rosenquist and Morrison, 1989). Thus, all treatments that reduce cluster compactness are expected to lead to a lower predisposition of clusters to early and severe incidence of pests and diseases (Molitor et al., 2012). Compactness also affects ripening homogeneity, the shaded berries tend to receive less solar radiation which affects berry composition and maturation dynamics (Pieri et al., 2016; Silvestre et al., 2017). Finally, loose cluster is a favorable factor for table grape appearance.

The compactness level of a cluster is the resultant of the sum variation of directly or indirectly related traits, linked to the rachis architecture or to the berry size. It is difficult to quantify, although there already exist indexes for the cluster compactness calculated from quantifiable parameters (Tello and Ibañez, 2014) as an alternative to the visual assessment recommended by the OIV (International Organisation of Vine [OIV], 2007). Recent studies in our group showed that, in a multi-cultivar frame, the number of berries and rachis dimensions are key components in the determination of cluster compactness, followed by berry dimensions (Tello et al., 2015). The final number of berries in the cluster is a consequence of the number of flowers in the inflorescence and their rate of conversion into berries (fruit set rate). These two traits are major responsible of the reproductive performance of a vine. All these mentioned traits are genetically determined, although some of them may be very influenced by environmental factors, leading to seasonal or individual variation (Dry et al., 2010). In grapevine, cultivars are vegetatively propagated, producing plants with the same genotype, but many of these cultivars have been maintained for several centuries, especially in those used for winemaking. For that reason, somatic mutations have accumulated in many plants of these varieties, allowing making clonal selection, where a single plant of the cultivar is multiplied to constitute a clone within the cultivar. Clones may differ in many traits, including cluster compactness and related traits, and constitute interesting material for genetic studies. In a previous work, clones from Garnacha cultivar showing variation in cluster compactness and other traits were compared at the gene expression level (Grimplet et al., 2017). Flowers from clones differing for the number of berries showed extensive differences in their transcriptome in the Garnacha cultivar at E-L 26 (cap-fall complete) and allowed identifying gene networks and genes potentially related to the phenotypical variation.

These previous results indicated that studying the grapevine reproductive performance was necessary for the identification of important genetic factors affecting cluster compactness. Specifically, we aim to determine the role of factors such as the initial number of flowers and fruit set rate, processes of the grapevine reproductive development that are under the control of plant hormones (Giacomelli et al., 2013). GAs mediate the formation of the inflorescence axis. Later cytokinins regulate the differentiation into flowers and are specifically needed for the growth of pistil (Pool, 1975), and flowering timing is controlled through the GA:cytokinin balance (Srinivasan and Mullins, 1981).

The goal of this work was to identify genetic and molecular processes behind the phenotypical differences between clones of Tempranillo cultivar differing in their reproductive performance and cluster compactness. To reach that goal we characterized clones at phenotipical, hormonal, and transcriptomics level and perform global analyses from these data. The final aim was to identify candidate genes and polymorphisms involved in the determination of flower number and fruit set rate in relation to cluster compactness.

## Materials and Methods

### Plant Material

Plant material was collected at Viveros Provedo’s plot in Logroño (La Rioja, Spain), where all the vines were treated in the same way. The four clones used in the analysis originated from a *Vitis vinifera* cv. Tempranillo clone collection grafted on Richter-110 rootstocks (Provedo Eguía et al., 2007). Two of the clones are described as producing compact clusters (“compact” clones: RJ51 and VP2) and two produce loose clusters (“loose” clones: VP25 and VP11) (Tello et al., 2015). Sampling was performed in 2012, 2014, 2015, 2016, and 2017 for phenotyping and in 2015 for hormones and RNAseq analyses, which were done on the same samples. These latest samplings were performed at E-L 13–14 (April 30), E-L 16–17 (May 14), E-L 18–19 (May 28), and E-L 26 (June 8) [developmental stages according to the modified E-L system for grapevine growth stages (Coombe, 1995)]. Flower samples were collected and immediately frozen in liquid nitrogen; once in the lab samples were kept at −80°C until their use.

### Phenotyping

Each clone was phenotyped during 2–5 years using morphological variables of the cluster and the berry related to compactness trait as described in Tello et al. (2015). In addition, the clones were characterized in 2016 and 2017 using new variables related to their reproductive performance, for which, 10 inflorescences from different plants were tagged and bagged before flowering. The bags were removed once all the calyptras (fused petals, one calyptra per flower) had fallen inside. Calyptras were scanned and counted to estimate the number of flowers. The same tagged inflorescences, already transformed in clusters, were collected at harvest stage for phenotyping. Basically, new variables consisted in counting data of the initial number of flowers existing in the inflorescence and of their derived organs in the ripe cluster: seeded (normal) berries, seedless berries, and live green ovaries (LGOs), also known as “hens,” “chickens,” and “shot berries,” respectively (Collins and Dry, 2009). These variables were used to estimate the fruitset rate (proportion of flowers that develop into berries – either seeded or seedless –), and the abnormal conditions named coulure (excessive proportion of desiccated or drop flowers) and millerandage (excessive proportion of post-flowering organs that develop into either seedless berries or LGOs) (Dry et al., 2010). Cluster compactness index CI-12 was calculated according to Tello and Ibañez (2014). Statistical comparisons between clones were done using SPSS v.24 (IBM, Chicago, IL, United States). PCA was performed with the R software package prcomp and visualized with the fviz_pca package.

### Hormones Analysis

Hormones were analyzed at the Servicio de Cuantificación de Hormonas Vegetales in the IBMCP in Valencia, Spain with a UHPLC-mass spectrometer (Q-Exactive, ThermoFisher Scientific) from at least 100 mg of flower and inflorescence material. Hormones included IAA, ABA, JA, SA, and the GAs GA_51_, GA_4_, GA_1_, G_29_, and GA_8_. Hormones were analyzed at E-L 13–14, E-L 16–17, E-L 18–19, and E-L 26 stages.

### RNA Extraction and RNAseq Analysis

Total RNA was extracted from samples using the Spectrum plant total RNA kit (Sigma ^1^) as recommended by manufacturer. DNase I digestion was carried out with the RNase-free DNase Set (QIAGEN). RNA integrity and quantity were assessed with a Nanodrop 2000 spectrophotometer (Thermo Scientific) and an Agilent’s Bioanalyzer 2100. RNA samples were processed to build strand-specific cDNA libraries (one per biological sample) using Illumina TruSeq RNA Library Preparation Kit (Illumina, San Diego, CA, United States). Sequencing of all 24 libraries (3 replicates ^∗^ 4 clones ^∗^ 2 stages) was conducted on two sequencing lane using Illumina HiSeq 2500 v4 platform (Illumina, San Diego, CA, United States) to produce 19–24 million strand-specific 125 bp paired-end reads per library. Sequencing was performed at the Center for Genomic Regulation (Barcelona, Spain).

Sequences analysis were performed using the Galaxy tool (Afgan et al., 2016) to streamline the process on the 24 samples. Reads were mapped to the reference (12X.V2) grapevine genome using TopHat 2.1.0 (Kim et al., 2013) allowing only for unique mapping and up to three mismatches per read mapped and a minimal quality of 20. The alignment was performed using the Grapevine reference annotation V.3 (Canaguier et al., 2017). Read counts were generated using featureCounts from Subreads 1.5.1 (Liao et al., 2013). Analysis of differential gene expression was performed using EdgeR (Robinson et al., 2010) between each pair of clones at the two time points. Gene expression clustering was performed using the Quality threshold (QT) clustering method (Heyer et al., 1999) complemented by hierarchical clustering (HCL) with a maximum distance threshold of 0.2. Clustering was performed with the TMEV software (Saeed et al., 2003).

### Sequence Polymorphisms Analysis

Detection of polymorphisms [SNPs and insertions/deletions (indels)] between clones was performed using the RNA-seq alignments bam files. Variant calling vcf files were obtained with the variant caller utility implemented in the SAMtools package v1.2 (mpileup, bcftool) (Li et al., 2009). The vcf files were filter for a quality >40 using vcffilter for the vcflib toolkit^2^. Other file handling operations were performed with vcftools (Danecek et al., 2011). 605 polymorphisms between clones were detected automatically with bftoolcall, with alleles appearing consistently in the three replicates per clone. For the purpose of this work, establishment of differences between the Tempranillo clones regarding their homozygosity/heterozygosity status followed strict rules. Only polymorphisms based on a minimal depth of 50 reads were considered. One clone was considered heterozygous for a given SNP when the number of reads of the minor allele represented at least 30% of all the reads for that locus and clone. The other clones were considered homozygous when contained up to one read for the minor allele, representing less than 2% of the reads. However, we only considered polymorphic site where no read was detected for the minor allele in at least one of the homozygous clones. All the remaining cases were left non-assigned. Only polymorphisms confirmed in all replicates after individual checking in IGV software (Thorvaldsdottir et al., 2013) were considered. The effect of detected polymorphisms considering grapevine 12X.V3 gene prediction was estimated using SnpEff v.2.0.3 (Cingolani et al., 2012).

### Functional Categories Analysis

To identify the biological functions over-represented within selected probe sets, functional enrichment analyses were performed using the Cytoscape plugin Bingo (*p <* 0.05) (Maere et al., 2005) adapted to the functional categories manually annotated described in Grimplet et al. (2012) updated for the differentially expressed genes absent in the previous annotation (v1).

## Results

### Phenotyping and Comparison of the Clones

Phenotypic analyses showed a large amount of variability within Tempranillo cultivar for many traits related to the reproductive development (Table 1). In several cases, the differences between clones are stable and robust as to be statistically significant after up to 5 years of data, with very different climatic conditions. Thus, the four clones displayed a consistent difference for the visually assessed cluster compactness between the two compact clones (RJ51 and VP2) and the two loose clones (VP25 and VP11). The compactness index CI-12 even showed significant differences between the four clones, with the same trend observed for the cluster weight, one of the index components, and for the number of seeds per berry, the other single variable for which all the four clones showed significant differences. It is generally accepted that seed content relates to berry size, but here only the compact clone RJ51 showed significantly larger berries. Rachis architecture does not seem to have a main role in the differences of cluster compactness between these clones, as key variables like the lengths of the cluster, of the first branch and of the second branch of the rachis do not differ significantly between the clones, while they were important in a multi-cultivar study (Tello et al., 2015). Only the cluster width, rachis weight, and number of nodes showed a differential behavior between compact and loose clones.

**Table 1:** Average phenotypic data from two, three, or five seasons data of the four Tempranillo clones under study.

	Number of seasons	*N*	RJ51	VP2	VP25	VP11	Total average
Cluster compactness (OIV 204)	5	205	7.00 a	6.52 a	3.78 b	3.23 b	5.06
Cluster compactness (CI-12)	5	204	1.07 a	0.92 b	0.72 c	0.59 d	0.82
Cluster weight (g)	5	204	429.06 a	361.41 a	255.89 b	207.47 b	309.40
Cluster length (cm)	5	204	19.51 a	20.11 a	18.35 a	18.37 a	19.05
Cluster width (cm)	5	204	12.17 a	12.17 a	10.78 b	10.44 b	11.35
Cluster No. flowers	2	69	297.38 b	395.25 ab	376.78 b	507.74 a	398.71
Cluster No. seeded berries	5	205	217.82 a	223.71 a	164.81 b	123.70 c	180.93
Cluster No. seedless berries	2	69	4.00 a	3.31 a	6.37 a	22.05 a	9.46
Cluster No. LGOs	2	69	19.47 a	28.19 a	31.16 a	17.32 a	24.12
Cluster fruitset rate	2	67	0.68 a	0.69 a	0.56 a	0.33 b	0.55
Cluster millerandage index	2	69	1.24 a	0.97 a	1.49 a	1.76 a	1.39
Cluster coulure index	2	67	2.53 b	2.48 b	3.75 b	6.38 a	3.94
Rachis weight (g)	5	204	19.04 a	19.33 a	13.67 b	11.53 b	15.74
Rachis No. nodes	2	79	22.42 a	23.25 a	19.05 b	18.75 b	20.85
Raquis length 1st branch (mm)	5	204	50.08 a	51.08 a	49.88 a	54.45 a	51.40
Raquis length 2nd branch (mm)	5	203	46.45 a	48.30 a	47.87 a	47.74 a	47.60
Berry weight (g)	3	119	1.94 a	1.49 b	1.53 b	1.62 b	1.64
Berry length (mm)	3	119	14.11 a	12.99 b	12.68 b	13.17 b	13.23
Berry width (mm)	3	119	14.66 a	13.54 b	13.09 b	13.45 b	13.68
Berry pedicel length (mm)	2	79	7.18 a	6.38 c	6.57 bc	6.92 ab	6.76
Berry No. seeds	3	119	2.58 a	2.31 b	1.81 c	1.38 d	2.01
Vine fertility	2	79	1.21 a	1.04 a	1.21 a	1.22 a	1.17

*The number of seasons and the total number of clusters (N) used for each variable is indicated. Within a row, a different letter after the mean value indicates significant differences at α = 0.05. OIV 204: Cluster compactness defined according to the “Organisation International de la Vigne et du Vin (OIV) descriptor number 204. LGO: Live Green Ovary.*

Finally, several variables related to the reproductive performance of the vine seem relate to compactness. Based on 5-year data, the number of normal (seeded) berries in the cluster was similar in the two compact clones, and significantly lower in the loose clones, where there is still a difference between VP25 and VP11, the clone with the lowest number. This is a reflection of the fruitset rates, with similar values for VP2 and RJ51, followed by VP25 and, with the lowest value, VP11. An inverse trend is observed for the number of flowers and coulure, where VP11 showed the highest value, although the differences between the other clones are not significant. Other variables showed no significant differences between clones (like Millerandage), or no relation with compactness trait (like the length of pedicel).

**FIGURE 1:**
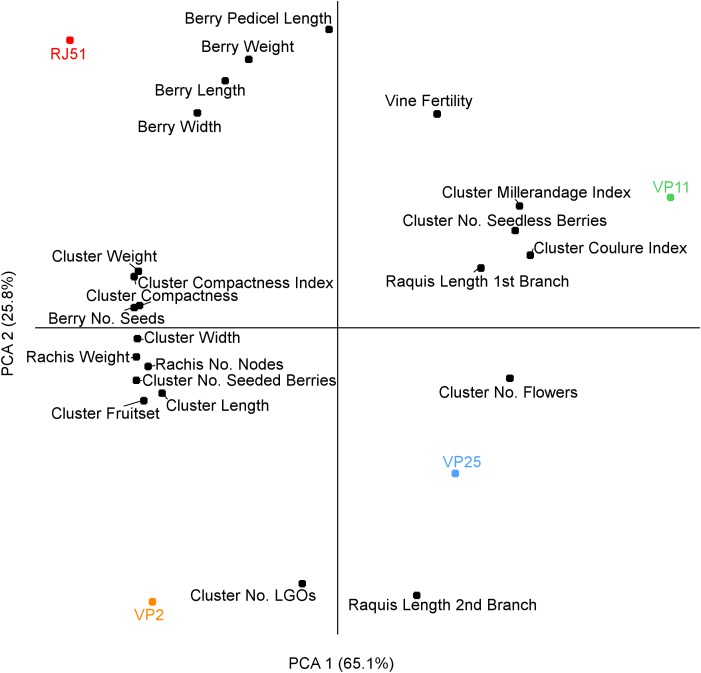
PCA analysis for the phenotype descriptors.

In order to evaluate the relation between each measured parameter and cluster compactness, we performed a principal component analysis (PCA) based on the phenotypic data (Figure 1). The PCA axis 1 separates factors correlating and anti-correlating with compactness, and separates the two compact clones, at the left, and the two loose clones at the right. The factors correlating the most with compactness were the number of seeds and cluster weight, on both components. Number of seeded berries, fruitset, rachis weight, number of rachis nodes and cluster width, and length also correlated with compactness. Results were in line with the results of Tello and Ibañez (2014) and Tello et al. (2015), including length of the first branch which anti-correlated with compactness. The other negatively correlating factors were number of seedless berries and its linked parameters millerandage and coulure index, as well as the only parameter that was not measured at the same stage as the compactness (at harvest), the initial number of flower. The second component of the PCA separates RJ51 from VP2 (compact) and VP11 from VP25 (loose) and distinguish variables less related to cluster compactness in these clones such as berry dimensions.

### Hormones Analyses

**FIGURE 2:**
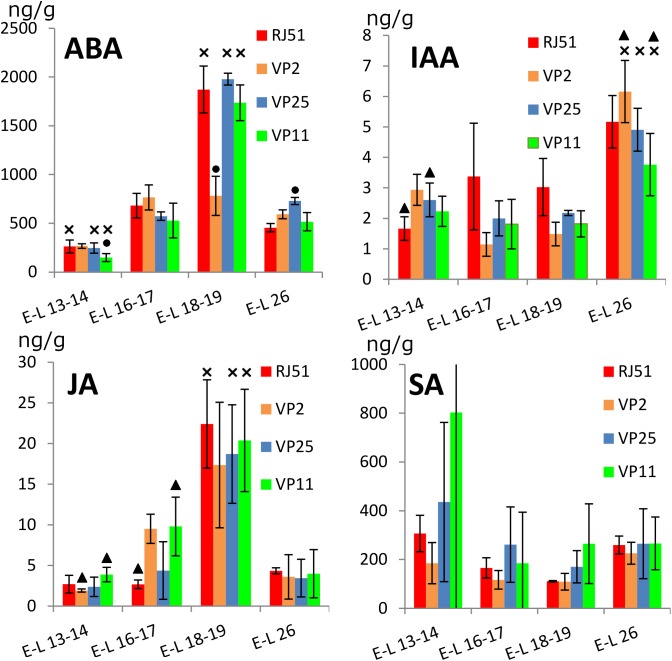
Abundance (ng/g fresh weight) of plant hormones within the grapevine flower development. ABA, abscisic acid; SA, salicylic acid; IAA, indol-3-acetic acid; JA, jasmonic acid. Cross, significantly different against the other three stages; circle, significantly different against the other three clones; and triangle, significant differences between two clones with different compactness.

Hormones profiles were obtained from samples taken every 2 weeks, at four different stages of the floral evolution, from E-L 13–14 when inflorescences started to be clearly distinguishable to E-L 26 at the end of flowering. The levels of hormones showed distinctive evolution patterns along flowering. Overall, ABA was significantly (*p* < 0.05) most abundant in E-L 18–19 (just before flowering) against the three other stages and significantly less abundant at E-L 13–14 against the three other stages (Figure 2). For individual clones, this pattern was true for RJ51, VP25, and VP11, only VP2 showed no significant differences between the three later stages, because at E-L 18–19 ABA was significantly less abundant in VP2 than in the three other clones. Besides, VP11 showed ABA levels significantly lower than the three other clones at E-L 13–14, while in VP25 ABA was significantly more abundant than in the three other clones at E-L 26.

Jasmonic acid profile was similar to that found for ABA: JA global levels were significantly (*p* < 0.05) higher in E-L 18–19 versus the three other stages, which showed no differences between them (Figure 2). The clones individually followed the same pattern, although in VP2 the difference between E-L 16–17 and E-L 18–19 was not significant. At E-L 13–14, JA levels were significantly more abundant in VP11 than in VP2 and VP25. At E-L 16–17, JA was significantly more abundant in VP2 and VP11 than in RJ51 and VP25.

Auxin (IAA) global levels were significantly higher at E-L 26, with no differences between the other stages (Figure 2). The clones individually mostly followed the same pattern; however, RJ51 did not show significant differences between E-L 16–17, E-L 18–19, and E-L 26, VP2 showed differences between E-L 13–14 and E-L 16–17, and VP11 did not show differences between E-L 13–14 and E-L 26. In the comparisons between the clones, RJ51 was the most different: at E-L 13–14 IAA was significantly less abundant in RJ51 than in VP2 and VP25; at E-L16–17, IAA levels were significantly higher in RJ51 vs. VP2, and at E-L 18–19, IAA in RJ51 was significantly more abundant than in VP2 and VP11. Besides, at E-L 26, VP2 levels were significantly more abundant than VP11.

Salicylic acid global levels did show no significant differences between the four stages, and the same occurred for the individual clones, except for RJ51 (Figure 2), where SA levels were significantly higher in E-L 13–14 and E-L 26 than in E-L 16–17 and E-L 18–19. No significant differences were observed between clones.

**FIGURE 3:**
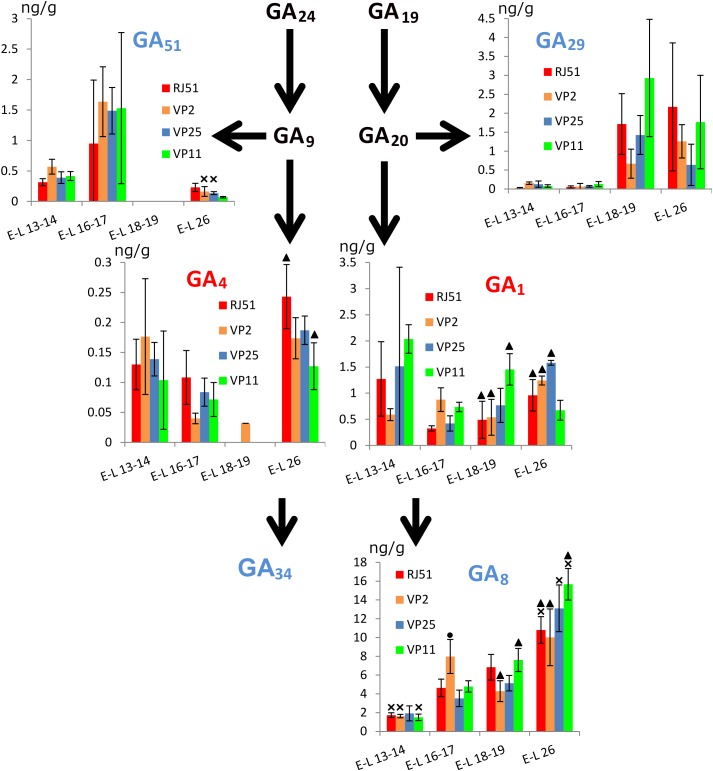
Abundance (ng/g fresh weight) of the main gibberellins within the grapevine flower development. Red, active gibberellins; blue, inactive gibberellins; black, intermediate. Cross, significantly different against the other three stages; circle, significantly different against the other three clones; and triangle, significant differences between two clones with different compactness.

Among the GAs analyzed, GA_51_ and GA_4_ were not detected at E-L 18–19. For active GAs (Figure 3), GA_1_ global levels were significantly more abundant at E-L 13–14 than at E-L 16–17, but with great disparity between clones. In VP2, GA_1_ levels were significantly higher at E-L 26 than at E-L 13–14 and E-L 18–19. The opposite was observed in VP11, GA_1_ was significantly more abundant in E-L 13–14 and E-L 18–19 than in E-L 16–17 and E-L 26. Comparing between the clones, GA_1_ levels at E-L 18–19 were higher in VP11 than in the other three clones, while at E-L 26, levels were significantly higher in VP25 than in RJ51 and VP11 and in VP2 vs. VP11.

Global levels of GA_4_ were significantly higher at E-L 13–14 and E-L 26 than in E-L 16–17. This pattern can be observed in all the clones. However, differences were significant only in RJ51 and VP25 between E-L 26 and E-L 16–17. Between clones, at E-L 16–17, GA_4_ levels were significantly higher in RJ51 than in VP2 and at E-L 26, in RJ51 vs. VP11.

For inactive GAs, GA_51_ showed the opposite pattern to GA_4_, with global levels significantly less abundant at E-L 13–14 and E-L 26 than at E-L 16–17. This pattern was observed in all the clones, although differences were significant only in VP2 and VP25. GA_29_ levels were significantly higher in the latest stages studied than in the earliest ones. This pattern can be observed in all the clones but significant results were only occasionally observed. GA_8_ is the inactivation product of GA_1_ and its global levels showed a steady increase over time with significant differences between all stages but between E-L 16–17 and E-L 18–19. RJ51, VP25, and VP11 followed this pattern; only in VP25, the difference between E-L 13–14 and the two intermediate stages and for VP2 between E-L 16–17 and E-L 26 were not significant. GA_8_ levels were higher at E-L 16–17 in VP2 than in the other clones, while at E-L 18–19 were more abundant in RJ51 and VP11 than in VP2. At E-L 26, again GA_8_ was more abundant in VP 11 than in the compact clones VP2 and RJ51.

In summary, considering all the clones together, significant differences between stages were found for all the hormones studied excepted SA, indicating their possible role during inflorescence growth and flowering. No differences (*p* < 0.01) were found in comparisons compact vs. loose clones, supporting the hypothesis that the mechanisms for loosening the clusters may be different in VP11 and VP25, and must be studied separately. The stages E-L 18–19 and E-L 26 were chosen for gene expression analysis because these two stages showed the most relevant differences between clones. Most remarkably, the higher abundance of GA_1_ in the loose clone VP11 at EL 18–19 discriminated it from both compact clones. VP11 also showed the lowest levels of the two active GAs at EL26, and the highest of GA_8_. VP25 only showed differential hormone levels with respect to the compact clones (and VP11) for ABA at E-L 26.

### Global View on Gene Expression Within the Clones

Principal component analysis (Supplementary File S1) showed that the replicates from each clone grouped together and shared similar expression profile. At both stages, the first dimension of the analysis grouped all the samples together (it represented 93.7% of the variance for E-L 18–19 and 85.4% for E-L 26). We performed the gene expression comparison on samples from only one cultivar in one organ at the same stage of development. Therefore, we expected that the expression of most genes was identical between conditions. As this first component presented little information, the components 2 and 3 were used for the plots. At E-L 18–19 (Supplementary File S1A), the second component of the PCA (method svd) discriminated compact clone RJ51 on one side and loose clone VP11 on the other, explaining 3.2% of the variance, we did not observed differences between VP2 and VP25 on this axis. The third component (1.1% of variance) separated VP25 from the three other clones, including VP2 contrary to the second component. At E-L 26, the second component (6.8% of variance) discriminated RJ51 from the three other clones (Supplementary File S1B). However, no component allowed discrimination in relation to compactness, the loosest clone VP11 was the closest to RJ51. The third component grouped together all the replicates of each clone and clearly separated VP2 from VP25.

### Gene Expression Profiles

After the visual global expression evaluation, we compared the expression for each gene among the clones in order to identify genetic evidences related to the phenotype differences. Gene differential expression detection was performed by pair-wise comparisons of clones at both developmental stages. A total of 1490 genes were differentially expressed between at least two clones at E-L 18–19 and only 168 at E-L 26 (Supplementary File S2). Of them, 28 genes maintained the same differential expression between clones in both stages while 26 other genes were differentially expressed in both stages but not with the same differences between clones. More than half of the differentially expressed genes were detected at E-L 18–19 in the comparison between the extremes clones in terms of compactness and reproductive performance parameters, RJ51 and VP11 (Table 2), in accordance to PCA results (Supplementary File S1). At E-L 26, the number of differential expressed genes was more evenly distributed among the comparisons, with overall little differences between clones (a maximum of 77 genes showed differential expression in any pair-wise comparison).

**Table 2:** Number of genes differentially expressed (fold change > 2, adjusted *p*-value < 0.05) at E-L 18–19 and E-L 26.

Comparison (1 vs. 2)	RJ51 vs. VP11	VP2 vs. VP11	RJ51 vs. VP25	VP2 vs. VP25	RJ51 vs. VP2	VP11 vs. VP25	Compact vs. VP11	Compact vs. VP25
Nb genes more expressed in 1 at E-L 18–19	378	118	100	9	18	167	245	2
Nb genes more expressed in 2 at E-L 18–19	838	284	241	9	5	159	79	6
Nb genes more expressed in 1 at E-L 26	16	27	14	12	31	7	4	1
Nb genes more expressed in 2 at E-L 26	10	16	57	11	46	19	1	5

To identify genes more probably related with the loose cluster phenotype, genes differentially expressed in each loose clone against the two compact clones together were detected (Table 2). In most of the comparisons, a small set of genes was found: five for VP11 vs. compact at E-L 26, eight for VP25 vs. compact at E-L 18–19, six for VP25 vs. compact at E-L 26. Only in the comparison compact vs. VP11 at E-L 18–19, a larger number of differentially expressed genes was detected (324), and allowed performing functional categories enrichment analysis.

Different types of gene expression profiles were found at both stages and were clustered as shown in Supplementary Files S3, S4. A larger number of clusters was found at E-L 18–19 than at E-L 26.

### Genetic Variation and Possible Effects on Gene Expression

We analyzed RNAseq data focusing in three kind of genes/differences that could be relevant for the study: transcripts with some polymorphism visible on the mRNA sequence irrespective of their expression level; genes that were only expressed or not expressed at all in one loose clone, in any of the two stages; and genes that seemed to express constitutively the same differences between clones in the two stages.

#### Sequence Polymorphisms in the RNAseq Data in Tempranillo Clones

Forty-seven genes showed some polymorphism among the four clones (Supplementary File S5) after the application of strict parameters for validation of polymorphisms and genotypes (homozygous/heterozygous). Additionally, these polymorphisms were validated on independent genome sequencing data for RJ51, VP11, and VP25 (data not shown). In a previous study (Royo et al., 2015), all the SNP fulfilling similar criteria were validated by PCR. Four of the polymorphisms were predicted to have a high putative impact (Table 3), likely leading to a non-functional allele in one of the clones. The whole expression observed for each of these four genes was not differential between the clones. They all code for proteins related to primary metabolism and cellular processes.

**Table 3:** Detected SNP and indels with a predicted high impact on the protein structure.

Putative impact	Sequence ID	Position	Affected clone	Polymorphism genotype status	Function
Stop gained	Vitvi14g00503	7926260	VP2	Heterozygous	Phosphoribosylaminoimidazole carboxylase
Stop gained	Vitvi17g00268	3076379	VP25	Heterozygous	Protein phosphatase 2C
Stop lost	Vitvi06g00376	4711553	VP25	Heterozygous	DNA-directed RNA polymerase III C1
Splice donor variant	Vitvi08g02244	16234252	RJ51	Heterozygous	High mobility group protein B1

#### Genes Absent or Present in One Loose Clone

The promoter areas of the genes are not visible through RNA sequencing, but expression patterns can show indications on the integrity of the promoter sequence. The most likely candidates for alteration of the promoter area in a specific loose clone are the genes that never exhibited expression in one loose clone, while being expressed in the others at the same stage and those that were expressed only in one loose clone (Table 4). Since these genes had raw ratios of expression that could tend to infinite (division by values close to zero reads), the EdgeR-corrected fold changes were high. Therefore, the 10 genes in the list had a fold change >8 at least.

**Table 4:** List of genes only expressed or never expressed in one loose clone at least in one stage (EL 18–19 or E-L 26) in RNAseq analysis.

		EL 18–19				EL 26			
Gene ID	Function	RJ51	VP2	VP25	VP11	RJ51	VP2	VP25	VP11
	**Only expressed in VP11**								
Vitvi13g02005	Subtilisin protease C1	−2.3	−2.0	0.1	**2.3**	0.0	0.0	0.0	0.0
Vitvi14g02553	Germin	−2.3	−0.9	−1.5	**2.3**	−2.0	−1.5	−**0.5**	**2.0**
Vitvi15g01183	No hit (Zinc finger)	−1.4	−1.0	−0.5	**1.4**	0.0	0.0	0.0	0.0
Vitvi15g01436	No hit (Yippee domain)	−1.4	−2.0	0.3	**2.0**	0.0	0.0	0.0	0.0
	**Absent in VP11**								
Vitvi02g01439	4 kDa proline-rich DC2.15	**1.8**	**2.0**	**1.6**	−2.0	0.0	0.0	0.0	0.0
Vitvi08g01534	Cytochrome P450 76A1	0.0	0.0	0.0	0.0	**0.9**	**1.1**	**1.9**	−1.9
**Vitvi13g02317**	Non-coding	**1.2**	**1.7**	**1.6**	−1.7	**1.0**	**2.2**	**2.3**	−2.3
	**Only expressed in VP25**								
**Vitvi02g01655**	No hit transposase	−1.6	−1.4	**3.0**	−3.0	−1.7	−2.6	**2.6**	−2.2
**Vitvi03g01791**	No hit transposase	−1.9	−2.2	**2.2**	−1.3	−0.9	−2.5	**2.5**	−1.1
**Vitvi11g01412**	Non-coding	−2.0	−2.3	**2.3**	−1.4	−1.2	−2.3	**2.3**	−1.1

*Numbers show gene expression as log2 ratio compared to the median value using EdgeR, in bold font when reads were detected in that clone, and in normal font when no reads were detected in that clone. Bold Gene ID, expression was consistent over stages.*

#### Genes With Constant Expression Along Flower Development

Additionally to the genes showing no expression, or only expression in one loose clone, we identified the genes that presented a stable differential expression between clones over both studied stages (Table 5) and with differences between a loose clone and the compact clones. Besides the four genes already reported in Table 4 (Gene ID in bold), we identified six other genes. These genes presented the same expression profile in both stages, with similar pair-wise differences between clones. The underlying hypothesis was that if a modification had occurred in the promoter sequence of a constitutively expressed gene, it would be visible in both stages. Among the detected genes, most of them were more abundant in loose clones. For many, their putative function revealed little information.

**Table 5:** List of genes with differential expression stable over stages between one loose vs. the other in RNAseq analysis.

		EL 18–19				EL 26			
Gene ID	Function	RJ51	VP2	VP25	VP11	RJ51	VP2	VP25	VP11
	**Over-expressed in VP11**								
Vitvi10g01862	Beta-amyrin synthase	−1.3	−1.1	−0.8	1.3	−0.9	−2.1	−1.6	2.08
	**Under-expressed in VP11**								
Vitvi04g01904	Serine hydrolase	1.15	1.3	−0.1	−1.3	0.78	0.96	−0.4	−1
Vitvi11g01170	Non-coding	−1.1	0.4	1.43	−1.4	0	1.12	1.35	−1.4
	**Over-expressed in VP25**								
Vitvi01g02019	Non-coding	−1.2	0.27	1.18	−0.2	−0.7	0.02	0.75	−0.7
Vitvi12g01876	Cupin region	−1	0	1	0.17	−0.8	−0.1	0.76	−0.4
Vitvi14g02595	No hit	−1.9	0.09	1.9	−0.3	−0.9	−1	1.04	−0.9

*Expression levels are log2 ratio compared to the median value.*

### Functional Analysis of the Differentially Expressed Genes

The previous analyses were directed to the identification of candidate genes for potential sources of variation on compactness-related traits; however, many other genes were differentially over-expressed in the study. Functional categories enrichment analysis was performed in order to identify the main mechanisms impacted in cluster compactness and their related traits at E-L 18–19 between VP11 and the two compact clones.

**FIGURE 4:**
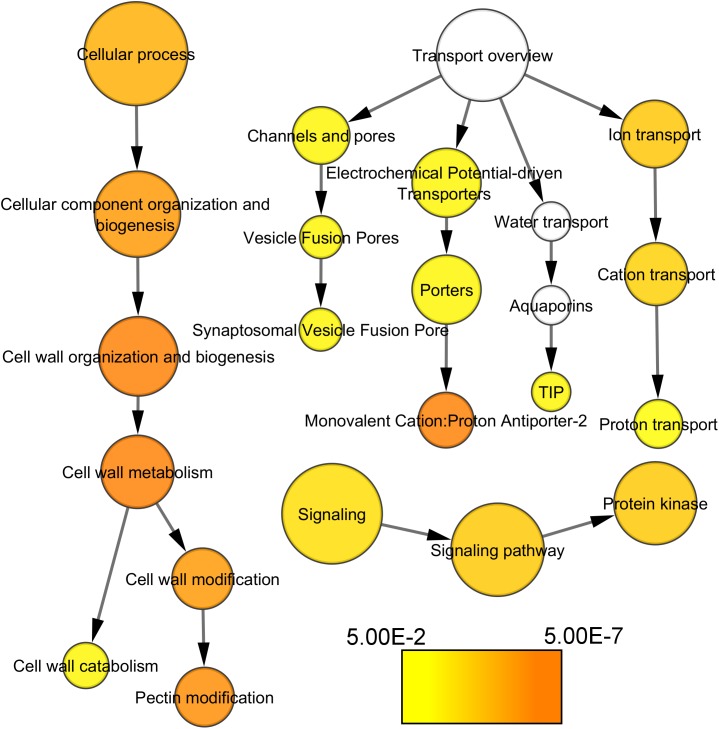
Functional categories of the genes significantly over-represented at E-L 18–19 in compact clones vs. VP11 (adj. *p*-value < 0.05). Colors are function of significance. White, not significant.

Most noticeably, cell wall-related functional categories were enriched in the compact clones, in particular in the process related to pectin modification (Figure 4). In addition, several transporters categories, such as proton transporter, monovalent cation–proton antiporter, TIP aquaporin, and synaptosomal vesicle fusion pores, and protein kinases. There is one category significantly under-represented in the list of genes more expressed in compact clones, the genes coding for proteins related to protein synthesis. It indicates that this process was remarkably stable between clones, allowing us to discard higher activity in protein synthesis as factor of the compactness.

**FIGURE 5:**
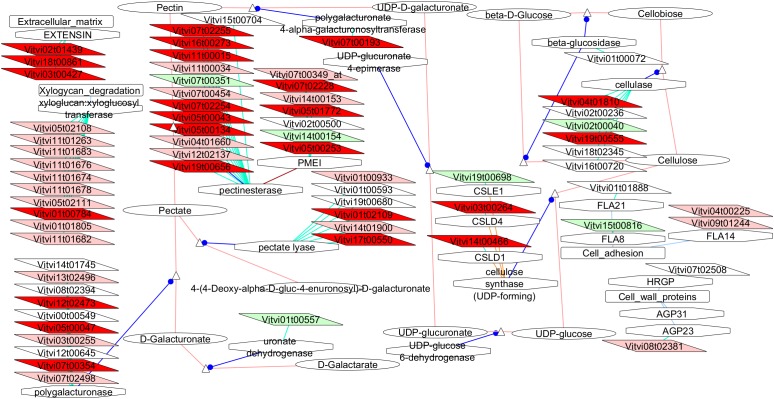
Adapted Cytoscape VitisNet networks including transcripts differentially expressed in flowers at E-L 18–19 between VP11 and the compact clones related to cell wall metabolism. Dark red, genes over-expressed in compact clones RJ51 and VP2; light red, genes over-expressed in compact clone RJ51 vs. VP11; light green, genes over-expressed in loose clone VP11 vs. RJ51. Figure was adapted from networks 40006 from Grimplet et al. (2009), genes in white background were over-expressed in another pairwise comparison; the transcripts not differentially expressed were removed from the picture.

The VitisNet representation of the events occurring in the cell wall metabolisms (Figure 5) highlighted also numerous changes specifically in the pectin metabolism-related genes with many isogenes under-expressed in VP11 vs. both compact clones and even more against RJ51 only.

**FIGURE 6:**
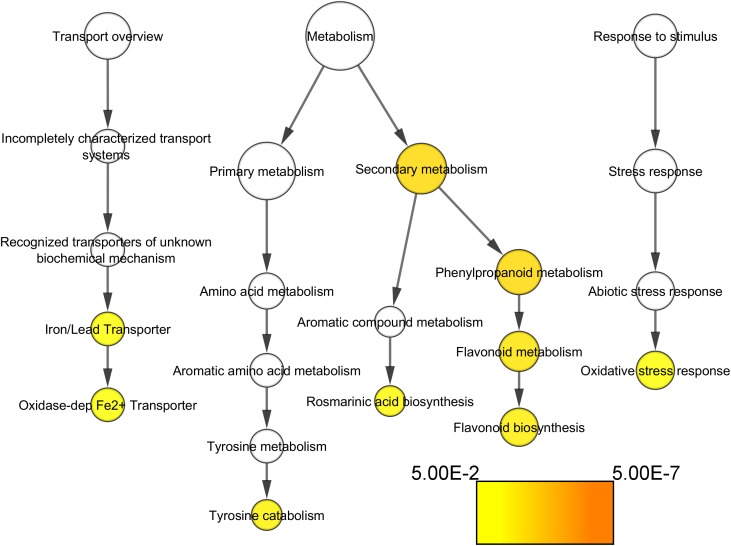
Functional categories of the genes significantly over-represented at E-L 18–19 in VP11 vs. compact clones (adj. *p*-value < 0.05). Colors are function of significance. White, not significant.

Concerning the functional categories over-represented in the VP11 clone vs. the compact clones, the categories related to flavonoids biosynthesis, oxidative stress response, and oxidase-dependent iron transporter (Figure 6) showed significant results.

**FIGURE 7:**
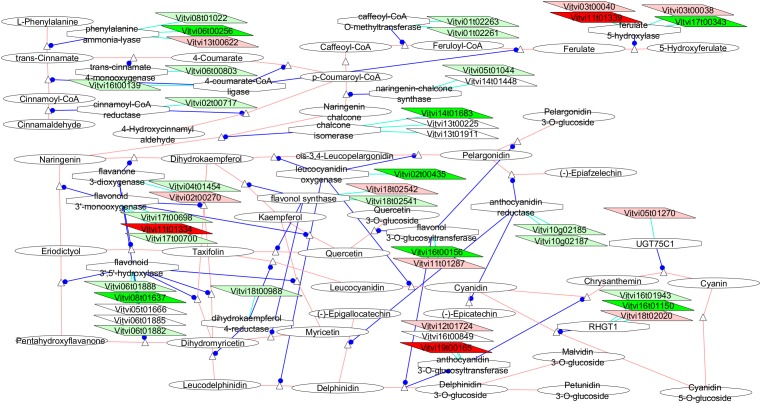
Adapted Cytoscape VitisNet networks including transcripts differentially expressed in flowers at E-L 18**–**19 between VP11 and the compact clones related to polyphenols metabolism. Dark red, genes over-expressed in compact clones RJ51 and VP2; light red, genes over-expressed in compact clone RJ51 vs. VP11; dark green, genes over-expressed in loose clone VP11 vs. both compact clones; light green, genes over-expressed in loose clone VP11 vs. RJ51. Figure was adapted from networks 10940 (phenylpropanoids), 10941 (flavonoids), and 10942 (anthocyanins) from Grimplet et al. (2009), genes in white were over-expressed in another studied condition; the transcripts not differentially expressed were removed from the picture.

The VitisNet representation of the networks related to the polyphenols (Figure 7) showed clear over-expression for most of the genes involved in the biosynthesis of the anthocyanin from the phenylalanine in VP11 vs. the two compact clones, and for VP11 vs. only RJ51.

## Discussion

The aim of this work was to identify genetic changes that affected genes involved in cluster compactness variation. For that, four different clones of the cultivar Tempranillo were studied, two of them (RJ51 and VP2) presenting compact clusters, as expected for the variety, and two presenting loose clusters (VP25 and VP11). Our hypothesis is that each of these two clones present loose clusters due to a genetic mutation originally produced in a single Tempranillo plant, which was vegetatively propagated. This mutation makes it differ from the normal plants with compact clusters but in a, basically, identical genetic background. The contrast in compactness was reproducible over the years and the clones were grown in the same conditions and parcel, at few meters from each other. Therefore, we expected that the differences between clones in phenotypic traits, hormones, and gene expression levels had a genetic origin that could be isolated by monitoring the gene expression and their polymorphism with little “noise” in the gene expression. Besides, in this work VP25 and VP11 have shown different phenotypic, hormonal, and gene expression characteristics, indicating that their loose phenotypes result from distinct mechanisms and should be studied independently.

### Phenotypical Differences and Hormone Levels Between Clones

Our previous findings showed that the two main components affecting the cluster compactness in a multi-cultivar frame were the cluster architecture and the number of berries (Tello and Ibañez, 2014; Tello et al., 2015; Grimplet et al., 2017). Within a single cultivar, Garnacha Tinta, only the number of berries showed stable differences between compact and loose clones (Grimplet et al., 2017). Therefore, in this work we undertook the cluster phenotyping with a special emphasis on traits affecting the reproductive performance of the selected clones and found significant differences in many of them. Some of these differential traits possibly related to cluster compactness such as the fruitset rate, the number of seeded berries per cluster, and the number of seeds per berry, which followed the trend compact clones > VP25 > VP11. The same tendency was observed for the pollen viability in these clones (Tello et al., 2018) and can explain these results, as a limited pollen viability may compromise pollination and fecundation, lowering the number of seeds per berry, the fruitset, and the number of seeded berries in the cluster.

For targeting the most appropriated phenological stages for the transcriptome evaluation, we first analyzed the hormones evolution during the inflorescence and flower development to pinpoint key stages with dramatic changes.

This work presents the first detailed study of the evolution of hormones levels during the formation of inflorescences and flowers. Several studies in grapevine have addressed the hormones levels from blooming but, to our knowledge, they were not measured in earlier stages. Giacomelli et al. (2013) analyzed the GAs quantities in flowers between the beginning and the end of flowering. They detected lower abundance at the end (E-L 26) than at the beginning of flowering (around E-L 19) for GA_1_, GA_4_ and GA_8_, which we only observed for VP11 for GA_1_. We could not relate this observation to the loose phenotype of VP11 because the authors performed the experiment on Pinot Gris clone R6, a somewhat compact clone. Interestingly, GA_ac_ applied on 1 cm inflorescence (around early E-L 13) induced an increase of flower number and branching (Khurshid et al., 1992) and we observed higher number of flowers in VP11, although less number of nodes.

Antolín et al. (2003) measured ABA from the beginning of flowering, which corresponded to the peak value they observed. It gradually decrease in later stages, which was also observed by Jia et al. (2013). For three of the four clones (all but VP2) we obtained similar results but additionally we observed that ABA showed a gradual increase earlier in the inflorescence development up to this point.

For IAA we also obtained similar results to those of Jia et al. (2013) on the overlapping studied stages but the higher amount of IAA at E-L 26 than at E-L 18–19 was not observed in such proportions. Before E-L 18–19, we observed no evolution of IAA.

Jasmonic acid in Arabidopsis is more abundant just before flowers open (Nagpal et al., 2005), as we observed in grapevine. Role of JA is to participate in the initiation of flowering, which is in accordance to our observation since it peaked at that stage.

### Potential Mutated Candidates for the Differential Phenotypes

Following our first goal, besides monitoring the global differences found in the molecular mechanisms affected within clones, we conducted analysis to identify the genes with variation between clones. The expression study through RNAseq allowed us to highlight three types of potential candidates: (i) Using the RNA sequences from the sequencing data, we could identify transcripts with SNP leading to modification of the protein integrity, such as the inclusion of a stop codon. (ii) Some genes showed a complete impairment of expression in at least one clone (no more than three detected reads), with possibly severe direct damage in their ability to be transcript due to variation in their regulator sequence. (iii) Other differentially expressed genes that showed identical expression between clones in both stages; their expression might not be influenced by environmental or physiological factors and the difference might be constitutive. We identified several genes fulfilling these criteria. However, functional analysis revealed that many of these three types of genes have an unclear or unknown function. It might mean that mechanisms important for compactness and related traits are yet to be studied and deciphered but we gathered some evidences for some genes of potential functional roles that can be discussed.

#### Possible Role of the Genes Containing Polymorphisms With High Impact on Protein

Among the 52 genes bearing variation between clones, four showed polymorphisms predicted to cause a high impact in the protein sequence. Vitvi14g00503 has clearly two polymorphic SNPs in VP2, one leading to a non-functional allele with a stop gain. This gene codes for a phosphoribosylaminoimidazole carboxylase but no evidence of a specific involvement in plant phenotype has been described in the literature.

Vitvi17g00268 has two polymorphic SNPs in VP25 (one non-functional with a stop gain). It corresponds to a protein phosphatase 2C that presented high expression in almost all tissues in the grapevine atlas in cv. Corvina (Fasoli et al., 2012), specifically in buds and was very and exclusively down regulated in pollen. The protein sequence contains an N terminal sequence of 70 aa that has not appeared in any other known plant proteins. It seems not to share specific homology to the phosphatase 2C proteins described as involved in ABA signaling (Rodriguez, 1998). Vitvi06g00376 has a stop codon loss in one allele in VP25. It corresponds to a DNA-directed RNA polymerase III subunit C1. No potential involvement in reproductive mechanisms was described in the literature. These two genes showed two different alleles in VP25 including one that may not be functional. The two proteins affected are involved in general cellular processes that if disrupted could affect compactness; however, their relation to compactness need to be more investigated.

Vitvi08g02244 is homologous to a high mobility group protein B1. One of the alleles in RJ51 has a splice-site donor variant leading to different 5′-UTR. Homologous genes have a described impact in the phenotype in other species. In Arabidopsis, mutant lacking HMGB1 had a slightly delayed and reduced germination rate, reduced root length, and enhanced sensitivity to methyl methane sulfonate (MMS) (Lildballe et al., 2008). However, we found two elements that might minimize its role in our study. The expression of the functional allele only, in terms of reads-count was equivalent to the expression in other clones, so the quantities of functional transcripts should be similar. In addition, this gene is not among the potential direct orthologs in grapevine of the Arabidopsis gene HMGB1 gene responsible for the mutant phenotype. Those were not differentially expressed here.

#### Genes Absent or Present Only in One Loose Clone

We used two strategies to highlight genes potentially presenting polymorphisms in their regulatory sequence. The first one was the identification of the genes only expressed or no expressed at all (less than three reads) in one loose clone in any stage. We hypothesized that promoters regions in those genes would be altered to reverse their expression in the affected clone.

Few of these genes presented a clear function (Table 4), none among those differentially affected in VP25. Vitvi14g02553 corresponds to a Germin, and its expression was absent in both compact clones, but was highly variable in the replicates of the loose clones. In the atlas, it was not present in flowers and was seed-specific, so one may wonder whether its expression in VP11 from the beginning to the end of flowering could disturb the normal pollination and fecundation processes. Germin-like proteins (GLPs) are involved in basal host resistance against powdery mildew (GER3 and GER4) in rice (Davidson et al., 2010). Pathogen tolerance is not directly related to compactness traits, but is well described that loose clusters show a reduced incidence of pests and diseases, attributed to their physical and physiological properties (reviewed in Tello and Ibáñez, 2018). There was no disease resistance genes correlating with that Germin. Another Germin co-express in CL02 (Supplementary File S3) with this gene indicating a possibility that a common Germin regulator might regulate its expression, and not being constitutively differentially expressed. Vitvi02g01439 was only expressed in E-L 18–19, except in VP11, for which no expression was observed. This gene codes for a 4-kDa proline-rich protein DC2.15, and it has been detected in a list of genes transiently expressed during early embryogenesis in carrot (Aleith and Richter, 1991). Its expression was also induced by the removal of auxins in carrot cell culture, but this cannot be correlated here, as there are no differences between VP11 and the other clones at E-L 18–19. Homologs of this protein were found in many species and it contains a plant lipid transfer protein/seed storage protein/trypsin-alpha amylase inhibitor domain but its role in-plant needs to be addressed. This gene belong to the CL19 (Supplementary File S3) of coexpression with three genes related to glycosyltransferases [one of cytokinins described later (Vitvi08g02412) and two of anthocyanin].

#### Genes With Constant Expression Along Flower Development

Most of the genes with differential expression between clones but not presenting variation between the two stages had an undefined or unclear function (Table 5). There were only three genes with homologs with a function described in other organism. The most interesting for the study of cluster compactness is Beta-amyrin synthase (Vitvi10g01862), which is constitutively more abundant in VP11. In the atlas, this gene was specific from buds, with low expression in the other organs. Beta-amyrin synthase is a key enzyme in the biosynthesis of the oleanolic acid that has anti-microbial activity (Liu et al., 2016). In addition to this gene and the above mentioned coding for a GLP, many genes related to pathogen resistance were detected as more abundant in loose clones in this study as well as in the previous one (Grimplet et al., 2017). These results indicate that lower pest and disease incidence in loose clusters might not be only related to physical features (Tello and Ibáñez, 2018) but also to molecular mechanisms favoring the tolerance. The previous study was performed on a different cultivar (Garnacha Tinta) with the same observation. It would be useful to study the effect of a lower degree of compactness on the expression of defense-related genes in different clusters of a single plant and at later stages.

### Cellular Mechanisms Working Differentially in the Clones

The genes described in the previous section exhibited dramatic differences of expression or sequence polymorphisms. Many other genes that were differentially expressed at a lower magnitude give invaluable information on the behavior of networks of genes involved in molecular mechanisms differentially affected in the studied clones. More specifically, we identified major changes in some hormones biosynthesis and signaling, mechanisms related to differences in the cell wall structure and the biosynthesis of flavonoids.

#### Hormones Metabolism and Signaling Play a Critical Role in Phenotypic Differences

Two of the analyzed hormones, JA and SA, will not be further discussed, as their quantities were stable between clones and we did not found in the expression analysis any element that would involve them.

##### Gibberellins

Gibberellins promote flowering through the activation of genes encoding the floral integrators in long-day plants such as Arabidopsis (Boss et al., 2004; Mutasa-Göttgens and Hedden, 2009). In grapevine, GAs inhibit flowering (Boss and Thomas, 2002) and GAs treatments performed at bloom tends to favor loosening and aeration of the clusters by reducing fruitset (Dokoozlian and Peacock, 2001) and berry number (Lynn and Jensen, 1966). Giacomelli et al. (2013) measured the abundance of different endogenous GAs in Pinot Noir flower development, including only one matching time point with our study: E-L 26. At this stage, our data showed one order of magnitude less that in that study, but all the ratios between the different GAs were conserved. Between clones, we observed differences in GAs levels, noticeably at E-L 18–19 a higher abundance of GA_1_ in VP11, the clone with a lower number of seeds, and Cheng et al. (2015) reported that a pre-bloom GA_ac_ treatment in a grapevine cultivar induced seed abortion. As mentioned before, GAs are known to play a role in flowering initiation but there was no difference of expression in all the known genes involved in the flowering pathway. We also never observed any difference between clones in flowering timing, which allowed us to discard the flowering regulatory network as a responsible event for the differences observed in cluster compactness or fruitset between clones.

Although the molecular mechanisms specific to grape responses to GAs are not fully known, some advances are in progress, and it has been recently shown that grape flower abscission mechanisms triggered by GA_ac_ application are different to those promoted by other stimuli, such as shading (C-starvation) (Domingos et al., 2015). In addition, Cheng et al. (2015) recently established the list of genes responding to a GA_ac_ treatment in the grapevine flower. We clearly identified changes in expression for the genes involved in the GA metabolism and genes known to be regulated by GAs, in particular genes related to cell wall mechanisms. Two isoforms (Vitvi09g00452 and Vitvi04g00435) of the enzyme catalyzing the last step of the active GA biosynthesis pathway, the GA 3-beta dioxygenase, were more abundant in the compact clone RJ51 than in the two loose clones at E-L 18–19, levels were always higher in VP2 too but difference is only significant with VP11 for Vitvi09g00452. Cheng et al. (2015) also documented an increase of GA 3-beta dioxygenase related to higher content in GAs, in that case after treatment with exogenous GA_ac_.

Hormones analysis at E-L 18–19 showed that, of the two active GAs, GA_1_ is more abundant in VP11 clone, while GA_4_ could only be detected in VP2. This might be explained by a higher turnover and rate of degradation. Three isoforms (Vitvi06g00659, Vitvi05g00163, and Vitvi19g02230) of the enzyme degrading active GAs into inactive GAs, the GA 2-beta dioxygenase, were differentially expressed. Cheng et al. (2015) found two GA 2-beta dioxygenase upregulated after GA_ac_ treatment, but they were other isoforms, on chromosomes 7 and 10. Vitvi06g00659 expression fitted the profiles of GA_1_ in clones and stages. At E-L 18–19, it was more abundant in loose clones (only significant between VP11 and RJ51) while a drop of expression in VP11 only was observed at E-L 26. At E-L 18–19, Vitvi05g00163 was also more abundant in loose clone VP11 but less abundant in VP2 than RJ51. Vitvi19g02230 is specifically down regulated in VP11 in E-L 18–19. These two genes did not show differences between clones at E-L 26.

One hypothesis is that expression might be part of an auto regulatory process induced by GA_1_. Such a process has been hypothesized before in maize and Arabidopsis (Phillips et al., 1995): GA-induced down-regulation of GAs biosynthetic enzymes. Here it might also involve GA-induced up-regulation of the GAs catalytic enzyme. Higher GA_1_ content correlates with lower biosynthetic GA3ox transcript level and higher catalytic GA2ox transcript level in VP11, possibly translated later in lower GA_1_ content in that clone at E-L 26.

The substrate specificity of some of the enzymes has been studied in grapevine by Giacomelli et al. (2013) for isoforms that were potentially involved in the catalysis of any active GA but no enzyme showed specificity for GA_1_. Vitvi05g00163, that was studied as (Vv)GA2ox4, was able to catalyze *in vitro* all 13-hydroxylated and non-13-hydroxylated GA substrates (GA_1_, GA_4_, GA_9_, and GA_20_). Vitvi19g02230 (GA2ox3) was able to catalyze active GA_1_ and GA_4_ (but not GA_9_ and GA_20_), as all the GA2ox studied.

Differences in GAs content may influence several mechanisms on which we observed different expression of involved transcripts. Exogenous GA_ac_ significantly increased wall extensibility in the wheat non-mutant controls but had no effect on the near-isogenic GA-insensitive genotypes (Keyes et al., 1990). We observed great changes of expression in many transcripts related to cell wall that will be discussed in a dedicated section below. Among the principal genes related to the cell wall and known to be regulated by GA, some were not differentially expressed in our study. GAs overproduction also promotes sucrose synthase expression and secondary cell wall deposition in cotton fibers (Bai et al., 2014). Vitvi09g00452 a sucrose synthase showed a higher expression in VP11, fitted to the profile of GA_1_ levels in this clone. The molecular basis for the long-known role of GAs in regulating cell expansion remains less clear, but recent work revealed that DELLA proteins, which are destabilized by GAs, interfere with the function of tubulin chaperones that are required for proper microtubule dynamics and consequently for cell expansion (Locascio et al., 2013); however, here no DELLA-coding genes showed differential expression.

##### ABA

We observed one main significant difference between clones for ABA levels, but it could not be related to cluster compactness and related traits: quantities of ABA were clearly less abundant in VP2 at E-L 18–19 compared to the others and were similar for all clones at E-L 26. ABA plays an antagonist role of GAs in the flower development by participating in the process of maintaining female organ in a dormant state before pollination in tomato (Vriezen et al., 2008). Consistently, after a steady increase, we observed a sharp decrease at end of bloom, once flowers are pollinated. The increase of ABA during the flower development was also observed in rose petal (Sood and Nagar, 2003) and in grapevine decrease in later stages of development (Owen et al., 2009). VP2, which presents a high number of flowers and berries, did not show this increase, so this possible disturbing of ABA over ovary dormancy does not seem to negatively affect flowering or fruitset. Unfortunately, little molecular evidences were gathered to correlate hormone levels and gene expression. One gene (Vitvi02g01286) related to the carotenoids metabolism involved in ABA biosynthesis showed expression differences, corresponds to VviCCD4a as described by Grimplet et al. (2014). It did not showed difference between VP2 and the loose clones and its expression was more abundant in RJ51 compared to the three other clones. Some genes known to be regulated by ABA were down regulated but neither showed differences between VP2 and the loose clones. One ABA receptor (Vitvi02g00695) was down regulated in VP11, only significantly with VP2, but values in RJ51 and VP25 are similar to VP2.

##### Auxin

Auxin plays a critical role in flowering, it is necessary for the initiation of floral primordia and flower formation (Cheng and Zhao, 2007). Auxin IAA was more abundant in the clone RJ51 in E-L 16–17 and E-L 18–19, important stages for the flower development. In our previous study, we highlighted the differences between clones in the expression of transcripts involved in the auxin transport, here none of these genes were differentially expressed. Still we observed changes of expression in genes known to be regulated by auxin, although many of them could be regulated by other hormones and might not be directly related to variation in auxin content between clones (Ren and Gray, 2015). The ARF6-like gene (Vitvi15g01767), highlighted in our previous study because it was the most differentially expressed, is not differentially expressed here. Instead, we observed an increased expression of nine SAUR genes that appeared localized in the same area of the chromosome 3. Vitvi04g01261 is a YUCCA flavin monooxygenase involved in the biosynthesis of auxin (Zhao et al., 2001) that was more expressed in RJ51 at E-L 18–19 compared with the three other clones. No relationship between cluster compactness phenotype and auxin levels or auxin-related gene expression could be established with the data gathered in this work.

##### Cytokinins

The role of cytokinins in flower development is unclear. Cytokinin content was not evaluated here and only one gene involved in the cytokinins metabolism is differentially expressed but it might have a significant impact. Vitvi08g02412, under-expressed in VP11 at E-L 18–19 is the closest grapevine homolog of the *Phaseolus* gene ZOG_PHALU, which is the only described *trans*-zeatin *O*-beta-D-glucosyltransferase in plants. This gene may regulate active vs. storage forms of cytokinins and was shown to have an impact on cell division and seed growth (Martin et al., 1999). Little is known on the disruption of the balance between *trans*-zeatin *O*-beta-D-glucoside and *trans*-zeatin but it may have an impact on the aforementioned processes too.

#### Cell Wall Metabolism and Flavonoids Metabolism Pathways Genes Expression Are Vastly Affected in VP11

##### Cell wall

Many genes related to cell wall metabolism were differentially expressed between clones at E-L 18–19. At the later stage E-L 26, differences of expression were mitigated, since only two genes from this network were differentially expressed. This indicates that cell wall modifications could be observed until beginning of flowering but key events might occur only early. Again, this timing of event supports the hypothesis that flowering itself probably has a minor or no role in the phenotypic differences observed. It also dismiss the possibility of the occurrence of specific mutations in late mechanisms promoting higher abscission of flower in loose clones. On another side, several elements depicted below indicate that key differences between VP11 and compact clones might be related to the gamete formation, specifically pollen. The pollen viability in these clones was evaluated by Tello et al. (2018) on plants from the same plot during the same season 2015, and also in 2017. Results were consistent and VP-11 showed the lowest pollen viability (around 60%), followed by VP25, while the two compact clones showed a pollen viability close to 100%.

The broad differential expression (24 genes differentially under-expressed in VP11 vs. both compact clones, plus 23 only against RJ51) indicates a lower activity in the cell wall formation in VP11. It is possibly related to disruptions in cellular multiplication in VP11, which is also confirmed by the higher expression of transcripts related to cytoskeleton regulation in compact clones. However, unlike at E-L 26 in Garnacha (Grimplet et al., 2017), the genes related to cell cycle are not differentially expressed. Many genes related to pectin metabolisms showed differential expression. Ten polygalacturonases were differentially regulated, as well as 12 pectinesterases and 6 pectate lyases. For the xyloglucan degradation, 16 xyloglucan endotransglucosylases (XET) were significantly more abundant in the compact clones. Cell wall metabolism in flowers has been studied, principally in the frame of the pollen formation, showing that disturbance of the pectin network integrity caused lower pollen viability in potato (Cankar et al., 2014) or cotton (Wu et al., 2015), which is coherent with our results. The other main topic related to cell wall is the pollen tube formation but the studied stages were not relevant in that case, since pollination occurs between E-L 19 and E-L 26. The main hypothesis is that the observed changes are related to pollen since one of the main differences between the two stages is the presence (E-L 18–19)/absence (E-L 26) of important amounts of pollen, as flowers sampled at E-L 26 had lost their anthers. Moreover, all the genes differentially expressed are described in the grapevine atlas as very abundant in pollen and flower (but not in petal and carpel) and most of them are even specific of flower and pollen (Fasoli et al., 2012). Polygalacturonases were shown to be abundant in pollen of corn and other grasses (Pressey and Reger, 1989), and they showed increased expression during pollen tube growth (Dearnaley and Daggard, 2001).

A high number of transcripts involved in the regulation of actin cytoskeleton were also observed as overregulated in compact clones at E-L 18–19 (none in E-L 26). This regulation also plays an important role in the pollen tube growth but little is known of its variation during earlier events in flower. A transcriptome analysis in *Arabidopsis* revealed that both cell wall metabolism and cytoskeleton were strikingly over-represented in pollen in preparation for the progamic phase, the pollen tube growth through the pistil (Honys and Twell, 2004). As for cell wall metabolism, the differentially expressed genes were detected as pollen specific in the atlas. As an example, cofilin is an actin depolymerizing factor (ADF), which were essential for pollen viability in *Physcomitrella patens* (Augustine et al., 2008). Two isogenes (Vitvi15g01148 and Vitvi16g01026) were over-expressed in compact RJ51 vs. loose VP11.

##### Flavonoids

At least one isoform of every gene in the phenylpropanoids metabolism from the phenylalanine to the anthocyanin was found differentially expressed and more abundant at E-L 18–19 in VP11 than in RJ51, and many of them also vs. VP2 and even vs. VP25 (Figure 7). Interestingly GA_ac_ treatment in grapevine flowers led to significantly higher polyphenol and anthocyanin content in wine (Teszlák et al., 2005). The much higher anthocyanin content also correlated to the lower *Botrytis* infection grade in GA_ac_-treated grapes in the same work. As VP11 showed higher GA_1_ content at E-L 18–19, it is reasonable to hypothesize that this increase in polyphenol metabolism gene expression might be related to GAs. Control of GAs over anthocyanin content has been documented but showed antagonist effect in different species and organs (Loreti et al., 2008). GAs were shown to be required for the induction of anthocyanin gene transcription in flowers of *Petunia hybrida* (Weiss, 2000) while in immature apple fruit, anthocyanin formation appears to be repressed by endogenous GA (Saure, 1990). Cheng et al. (2015) also detected enrichment of the phenylpropanoids metabolism category after GA_ac_ treatment of grape flowers. Lower disease incidence in loose clusters, when looseness is related to higher GA content as for VP11 (but not VP25), might be favored as a side effect by an overproduction of phenylpropanoids and/or anthocyanin, likely since flowers stages. Studies done on the polyphenols contents in musts produced from these clones did not show the same tendency, and VP11 showed the lowest values of anthocyanins and total polyphenols [(Provedo Eguía et al., 2007) and personal communication]. So, the protective effect against pests and diseases through the activation of phenylpropanoid network would occur during early flowering, perhaps limiting the infections or infestations at this time, and would contributed later, together with other physical and physiological attributes, to lower the incidence of diseases of loose clusters.

## Conclusion

We described here for the first time in grapevine intra-cultivar differences at three levels: phenotypical, hormonal, and transcriptional, among four clones, two producing compact clusters and two producing loose clusters. Evolution of hormonal levels during inflorescence development have been shown in Tempranillo, with clear differences between hormones and few differences between the clones. Considering all the analyses, loose clone VP25 presented few differences with the compact clones, giving no clues about general mechanisms or gene networks involved in its loose phenotype. Although several differentially expressed or polymorphic genes versus compact clones might be involved in looseness, even though their role is currently unknown. On the contrary, clone VP11 showed large differences with the compact clones in several aspects, mainly phenotypical and in gene expression. Stage E-L 18–19, corresponding to the start of flowering, was the most informative stage, and gave some indications based on the coincidence of higher levels of active GA_1_ and reduced expression of genes involved in cell wall metabolism. As in VP25, a number of genes differentially expressed could also play a role in the phenotypical differences and are worthy to be further investigated.

## Author Contributions

JI and JG designed the study and drafted the manuscript. JG, SI, JT, EB, and JI performed sampling and phenotyping. JI performed phenotypic analysis. JG performed the hormonal and gene expression analysis and interpretation. All authors read and approved the final manuscript.

## Conflict of Interest Statement

The authors declare that the research was conducted in the absence of any commercial or financial relationships that could be construed as a potential conflict of interest.

## Footnotes

1 www.sigmaaldrich.com

2 https://github.com/vcflib/vcflib

## Supplementary Material

FILE S1 **A)** Principal component analysis at E-L 18–19 with the differentially expressed genes of all replicates of the four Tempranillo clones. **B)** Principal component analysis at E-L 26 with the differentially expressed genes of all replicates of the four Tempranillo clones.

FILE S2 List of significantly differentially expressed genes (twofold ratio, *p*-value < 0.05) between at least two clones at E-L 18–19 or E-L 26.

FILE S3 Clustering of the differentially expressed genes at E-L 18–19 using QT+HCL method with a threshold of 0.2. Red background: lower expression in VP11, green higher expression in VP11, blue: higher expression in VP25 vs. compact clones. Cluster 30 corresponds to leftover genes that did not fit any of the profiles.

FILE S4 Clustering of the differentially expressed genes at E-L 26 using QT+HCL method with a threshold of 0.2. Red background: lower expression in VP11, blue: higher expression in VP25 vs. compact clones. Cluster 14 corresponds to leftover genes that did not fit any of the profiles.

FILE S5 List of variant polymorphisms between clones. Dark color: homozygous SNP, light color: heterozygous SNP. White: unclear polymorphism.

## Abbreviations

ABA: abscisic acid
GA: gibberellin
GA_ac_: gibberellic acid
IAA: indole-3-acetic acid
JA: jasmonic acid
SA: salicylic acid
SNP: single-nucleotide polymorphism.
