# Supplementary figures and images for: Phenotypic, Hormonal, and Genomic Variation Among Vitis vinifera Clones With Different Cluster Compactness and Reproductive Performance
Source: Front Plant Sci. 2019 Jan 7;9:1917. doi: 10.3389/fpls.2018.01917 (PMC6330345; doi:10.3389/fpls.2018.01917)

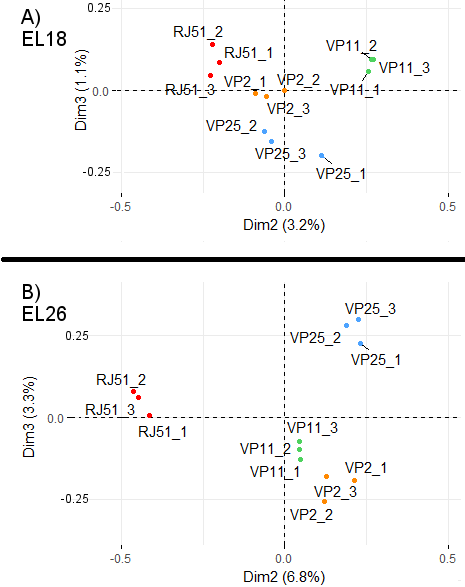

Supplement: FILE S1 — A) Principal component analysis at E-L 18–19 with the differentially expressed genes of all replicates of the four Tempranillo clones. B) Principal component analysis at E-L 26 with the differentially expressed genes of all replicates of the four Tempranillo clones. [file Image_1.TIFF]

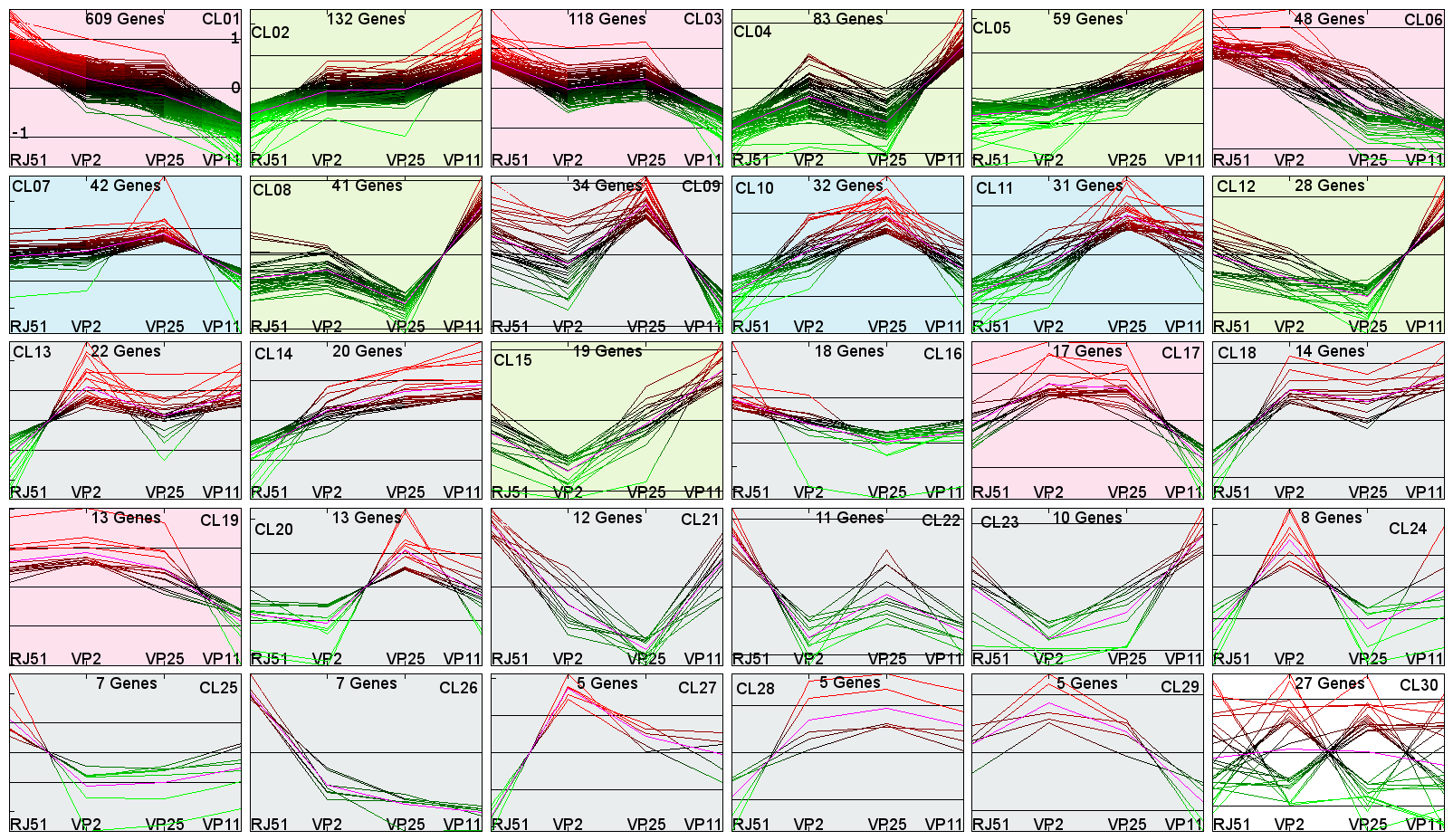

Supplement: FILE S2 — List of significantly differentially expressed genes (twofold ratio, p-value < 0.05) between at least two clones at E-L 18–19 or E-L 26. [file Image_2.TIFF]

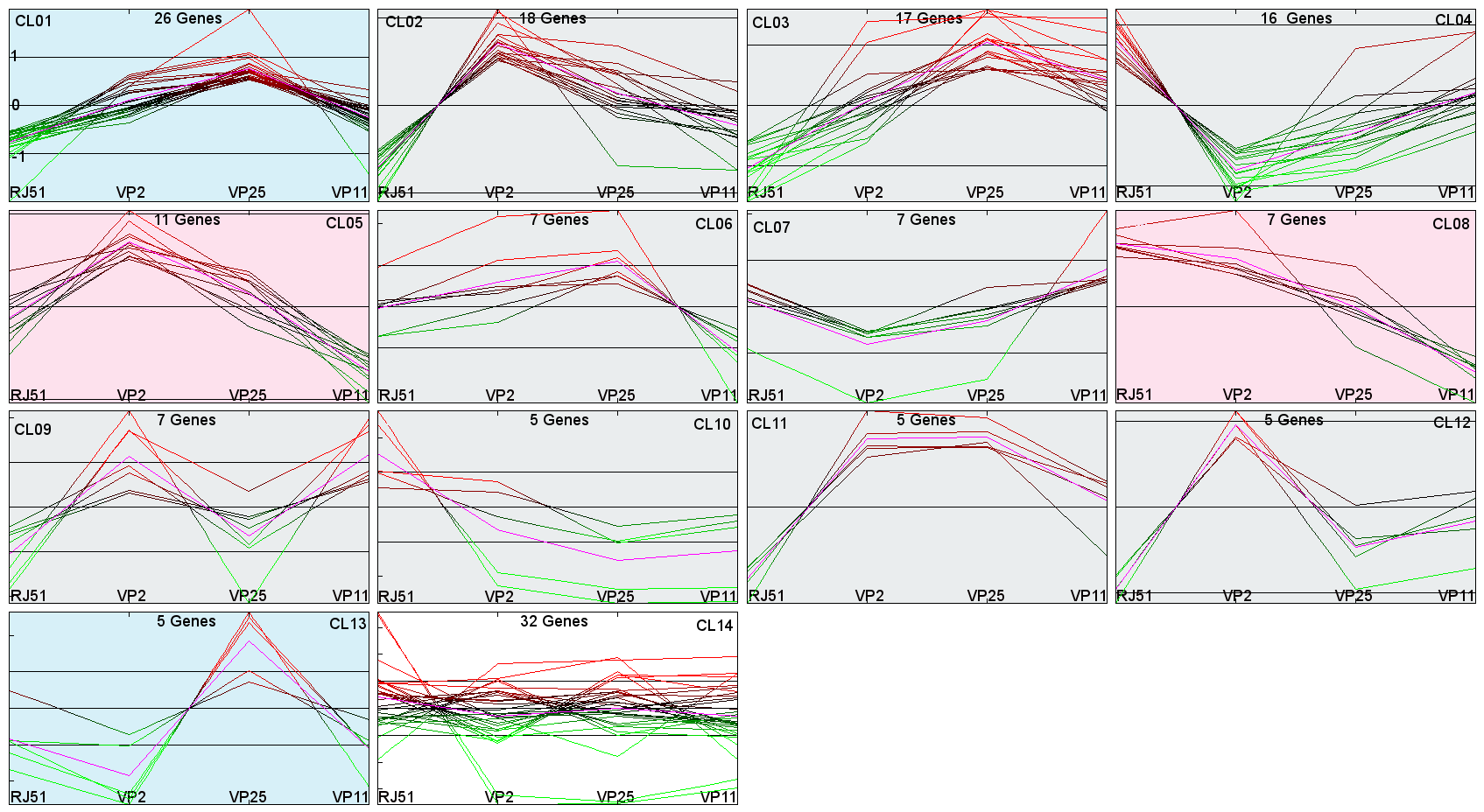

Supplement: FILE S3 — Clustering of the differentially expressed genes at E-L 18–19 using QT+HCL method with a threshold of 0.2. Red background: lower expression in VP11, green higher expression in VP11, blue: higher expression in VP25 vs. compact clones. Cluster 30 corresponds to leftover genes that did not fit any of the profiles. [file Image_3.TIFF]
